# Acute nutritional ketosis during early recovery from aerobic exercise does not affect skeletal muscle transcriptomic response in humans

**DOI:** 10.1007/s00421-025-05987-9

**Published:** 2025-09-17

**Authors:** Erick Mosquera-Lopez, Julien Louis, Jason P. Edwards, Jamie Pugh, Mark R. Viggars, Daniel J. Owens, Jose L. Areta

**Affiliations:** 1 Research Institute for Sport & Exercise Sciences (RISES), Liverpool John Moores University, Tom Reilly BuildingByrom St Campus, Liverpool, L3 3AF UK; 2 Department of Physiology and Aging, University of Florida, Gainesville, FL USA

**Keywords:** Ketone supplementation, Aerobic exercise, Gene expression profiling, Glycogen metabolism, Erythropoietin

## Abstract

**Purpose:**

Nutritional ketosis is purported to enhance skeletal muscle recovery and adaptation to exercise, yet precise adaptive mechanisms are unknown. We investigated the post-exercise molecular response to ketone monoesters (KME) in skeletal muscle by characterising the early transcriptomic response.

**Methods:**

Following a randomised, double-blind, crossover design, recreationally active men (n = 9, age: 26 ± 5 (means ± SD) y; V̇O_2max_: 47 ± 4 mL·kg^−1^·min^−1^) completed two experimental trials where they ingested either 1.25 g·kg^−1^ of KME or a taste-matched placebo (PLA) drink during exercise (90-min cycling at 60% of V̇O_2max_) and 3-h recovery. Blood samples were taken throughout for hormone and metabolite analyses, and muscle biopsies were taken at baseline and 3 h post-exercise for glycogen and genome-wide gene expression analyses.

**Results:**

Recovery ßHB concentrations were higher in KME (4.1 ± 0.7 mM) vs PLA (0.1 ± 0.0 mM, *P* < 0.001). Erythropoietin (EPO) showed a main effect of time (*P* = 0.044), but no condition effect (*P* = 0.087) or interaction (*P* = 0.318). Skeletal muscle glycogen decreased post-exercise (−57%, *P* < 0.001) as expected, but showed no condition effect (*P* = 0.889) or interaction (*P* = 0.907). We measured the expression of 16,898 genes, and despite a clear time effect on the skeletal muscle transcriptome (1561 differentially expressed genes post vs pre-exercise; *q* < 0.05 fold change > ± 1.5), there was no effect of condition.

**Conclusions:**

KME did not demonstrate an effect on EPO concentration, muscle glycogen or transcriptome, suggesting DNA translation is likely not a process directly regulated by acute ketonaemia that increases early post-exercise.

**Supplementary Information:**

The online version contains supplementary material available at 10.1007/s00421-025-05987-9.

## Introduction

To improve performance and maximise adaptation to training, athletes make use of the latest available ergogenic aids to seek an edge over their competition. One such novel ergogenic aid is ketone monoesters (KME), which have recently been developed as an effective intervention to induce high levels of acute nutritional ketosis (Clarke et al. 2012), defined as the rapid increase in ketonaemia by nutritional means (Poff et al. 2020). KMEs had initially shown promise as an additional metabolic fuel source during exercise to improve physical performance in athletes (Cox et al. 2016). Nonetheless, direct assessment of ketone oxidation during exercise has shown to be too small to provide a meaningful contribution of oxidisable substrate to improve performance (Dearlove et al. 2021), and KME supplementation has failed to show enhancement of endurance performance in most research studies (Brooks et al. 2022). However, the metabolic, endocrine and physiological responses triggered by increased ketonaemia through nutritional ketosis may represent a stimulus that could positively affect adaptation to exercise, particularly in skeletal muscle, and pose KME as a potential ergogenic aid to optimise recovery (Robberechts and Poffé 2023).

KME supplementation has shown to improve training adaptation and recovery (Holdsworth et al. 2017; Poffé et al. 2019). KME supplementation enhanced recovery in trained males undergoing a 3-week intensified training intervention, reflected in a reduction of symptoms of overtraining and an improvement in endurance performance (Poffé et al. 2019). Although the mechanisms behind these observations were unclear, recent research showing an increase in erythropoietin (EPO) by KME suggests that EPO may play a role in modulating the adaptive response to overtraining (Evans et al. 2022). Indeed, a secondary analysis of the 3-week overtraining study (Poffé et al. 2019) revealed that KME increased blood EPO concentrations, along with increased skeletal muscle VEGF messenger ribonucleic acid (mRNA) expression and protein content and capillarisation of skeletal muscle (Poffé et al. 2023b). In addition, post-exercise glycogen resynthesis has been hypothesised to be accelerated with KME supplementation (Holdsworth et al. 2017), but results from human studies are still inconclusive (Mansor and Woo 2021). Therefore, despite the promising potential of KME for skeletal muscle recovery (Robberechts and Poffé 2023), the cellular mechanisms driving the response remain uncertain.

A hallmark of exercise and diet interventions is the extensive modulation of gene expression of skeletal muscle in the early post-exercise period (Pillon et al. 2020; Rowlands et al. 2011; Pilegaard et al. 2005). This, therefore, represents an important biological point of analysis to determine the physiological effects of KME in skeletal muscle. Indeed, research suggests that ßHB could potentially regulate the skeletal muscle transcriptome through post-translational mechanisms related to the epigenetic regulation of histone proteins in HEK 293, HMEC-1, and L6 myotubes (Xie et al. 2016; Huang et al. 2021; Chriett et al. 2019), and the phosphorylation state of key skeletal muscle signalling proteins linked to transcriptional factors or co-activators in humans (Vandoorne et al. 2017; Poffé et al. 2023a). However, evidence in humans is restricted to the effects of KME on post-exercise targeted mRNA expression such as vascular endothelial growth factor (VEGF) and endothelial nitric oxide synthase (eNOS) after a 3-week intervention (Poffé et al. 2023b), and the only research examining the effects of ketone bodies on the transcriptome is derived from in vitro and animal models (Ruppert et al. 2021; Chen et al. 2022; Roberts et al. 2022), which are limited to study designs with no exercise (Ruppert et al. 2021; Chen et al. 2022) and in tissues other than skeletal muscle (Huang et al. 2021). No studies to date have investigated the influence of KME supplementation during early recovery from exercise on skeletal muscle transcriptomic response in humans.

With this in mind, we aimed to explore for the first time the direct effect of KME on skeletal muscle during early recovery from aerobic exercise with a comprehensive genome-wide assessment of the transcriptome utilising state-of-the-art methodology for mRNA sequencing and establish the modulation of molecular pathways regulated in human muscle by KME. We hypothesised that KME would have a marked modulatory effect on skeletal muscle transcriptome and increase EPO concentrations, as well as muscle glycogen resynthesis during recovery.

## Methods

### Participants

We recruited 10 recreationally active (≥ 3 endurance-based exercise sessions per week, V̇O_2max_ ≥ 40 mL·kg^−1^·min^−1^), healthy, non-smoking males. One participant was excluded from the final analysis due to technical problems (error in provision of experimental drinks, discovered retrospectively upon blood βHB assessment), with the final dataset including 9 participants (age: 26 ± 5 (means ± SD); height: 1.80 ± 0.07 m; body mass: 80 ± 9 kg; V̇O_2max_: 47 ± 4 mL·kg^−1^·min^−1^). Participants provided written consent, and the study was approved by the local Research Ethics Committee of Liverpool John Moores University (ref. no. H23/SPS/043).

### Preliminary testing

Participants completed two preliminary sessions within 1 week before the start of the first condition. During the first visit, subjects performed a maximal incremental exercise test on a bicycle ergometer (Corival CPET, Lode B.V, Groningen, The Netherlands), where the initial workload was set at 125 W, followed by 25-W increments per min, until exhaustion (Bishop et al. 1998). Respiratory gas exchange was measured continuously during the test using a Vyntus™ CPX metabolic cart (Vyaire, Mettawa, Illinois, USA) and the highest oxygen uptake measured over a 30-s period was defined as V̇O_2max_. During the second session, participants were familiarised with the exercise testing procedure that consisted of 45 min at 60% V̇O_2max_.

### General study design

**Fig. 1:**
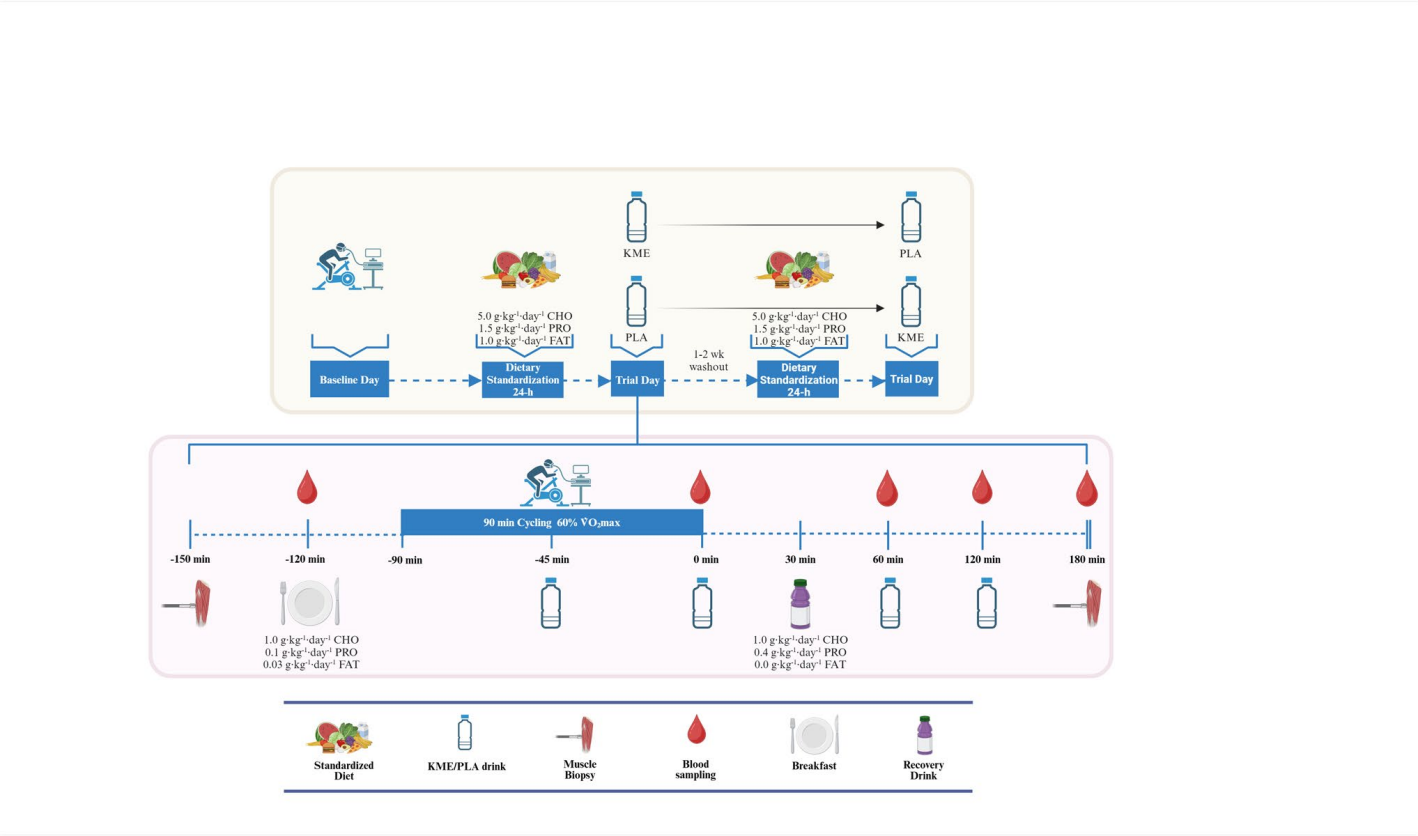
Schematic of the study protocol. The study involved two experimental sessions following a randomised, double-blind, crossover design, with a washout of 7–14 days in between. During each visit, subjects completed a 90-min steady state cycling at 60% V̇O_2max_. Participants consumed four doses of a KME or PLA drink ingested at min 45 of exercise and at 0, 60, and 120 min post-exercise. Breakfast and post-exercise recovery drink were provided 30 min pre-exercise and 30 min post-exercise, respectively. Venous blood samples were obtained 30 min pre-exercise and throughout the post-exercise recovery period. Muscle samples were obtained 60 min pre-exercise and 180 min post-exercise

An overview of the study design is shown in Fig. 1. This randomised, double-blind, placebo-controlled, crossover design study involved two experimental sessions involving two experimental visits, separated by 7–14 days. Each experimental session comprised a 90-min cycling session at 60% V̇O_2max_. Subjects received either 1.25 g·kg^−1^ (range: ~ 85–110 g) of KME [> 96% (*R*)-3-hydroxybutyl (*R*)-3-hydroxybutyrate] or a volume and taste-matched placebo (PLA) drink. Both drinks were spread in four dosages (0.5 g·kg^−1^, 0.25 g·kg^−1^, 0.25 g·kg^−1^, 0.25 g·kg^−1^) to be ingested at min 45 of exercise and at 0, 60, and 120 min post-exercise.

### Experimental sessions

Forty-eight to twenty-four hours before each experimental session, participants were free to follow their usual food intake and exercise habits, and any exercise performed prior to the first trial was recorded and replicated prior to the second trial. Twenty-four hours before each experimental session, subjects were instructed not to consume caffeinated drinks, abstain from exercise or strenuous physical activity, and were provided a custom-made, prepackaged diet containing 5 g·kg^−1^·day^−1^ carbohydrate (CHO), 1.5 g·kg^−1^·day^−1^ protein (PRO), and 1 g·kg^−1^·day^−1^ fat (FAT).

On trial days, participants arrived at the laboratory in the morning (0730 to 0830 h) and venous blood samples were drawn at 30 min pre-exercise and 0, 60, 120, and 180 min post-exercise. Subjects received a standardised breakfast containing 1 g·kg^−1^ CHO, 0.1 g·kg^−1^ PRO, and 0.03 g·kg^−1^ FAT exactly 30 min pre-exercise, and at 30-min post-exercise, participants consumed a recovery drink containing 1 g·kg^−1^ CHO and 0.4 g·kg^−1^ PRO. During the exercise, respiratory gas exchange (Vyntus™ CPX metabolic cart, Vyaire, Mettawa, Illinois, USA), heart rate monitor (H10 Polar, Polar Electro Oy, Kempele, Finland), and rating of perceived exertion (RPE, 6–20 scale, (Borg 1982) were also recorded in the last 3 min of every 15 min, and finger-prick blood samples were collected to measure lactate concentrations (Biosen C-Line, EKF Diagnostics, Cardiff, UK).

### Ketone and placebo drinks

KME drinks were composed of water, KME (TdeltaS Ltd, Thame Oxfordshire, UK), 5% w/v sucralose (MyProtein, Northwich, UK), and 1% v/v strawberry flavour drops (Myprotein, Northwich, UK). PLA drink contained water, bitter sucrose octaacetate (Sigma-Aldrich, Bornem, Belgium), 5% w/v sucralose, and 1% v/v strawberry flavour drops, dissolved and in proportion to water volume. Drinks were provided in opaque bidons, and double-blinding and participant order consumption randomisation of KME and PLA drink was performed by an investigator who was not involved in the study.

### Venous blood sampling and analysis

An indwelling cannula was inserted into the antecubital vein of one arm [Becton Dickinson (BD) Nexiva Closed IV Catheter, Becton Dickinson U.K. Limited, Wokingham, UK)], and samples were collected in blood collection tubes (EDTA, lithium heparin and serum, BD Vacutainers, Becton Dickinson U.K. Limited, Wokingham, UK). Tubes were centrifuged (3000 rpm for 10 min at 4 °C), and the supernatant was stored at − 80 °C until later analysis. Serum EPO was assessed in triplicate using commercially available enzyme-linked immunosorbent assay (ELISA, ab274397 Human Erythropoietin SimpleStep ELISA kit, Abcam), whereas serum βHB and glucose were measured in duplicate using the RX Daytona + (Randox Laboratories, Crumlin, UK: assay codes RB1007 GL8319, respectively). EPO intra-assay coefficient of variation was 4.9 ± 2.2%. βHB and glucose intra-assay coefficient of variation were 3.2 ± 4.2% and 1.7 ± 1.8%, respectively.

### Muscle biopsy

Skeletal muscle biopsies were obtained from the vastus lateralis of the quadriceps ~ 60 min pre-exercise and 180 min post-exercise. Each trial day, biopsies were taken from the same leg through different incisions. Anaesthetic (0.5% Marcaine) was applied prior to sampling, and a Conchotome biopsy needle was utilised to obtain muscle samples. Immediately, muscle samples were washed with phosphate buffered saline, and non-muscle material was removed. Muscle samples were instantly snap frozen in liquid nitrogen and stored at −80 °C until further analysis.

### Glycogen assay

Muscle glycogen concentration was determined according to the acid hydrolysis method using freeze-dried muscle samples that were dissected under the microscope to eliminate impurities of blood and connective tissue (Van Loon et al. 2000; Doering et al. 2019). Glucose concentration was quantified with the hexokinase method using a commercially available kit (GL8319; Randox Laboratories, Crumlin, UK), and glycogen concentration (mmol·kg^−1^ dry mass) was then calculated. Samples were analysed in duplicate and intra-assay coefficient of variation was 10.0 ± 8.9%.

### RNA isolation and RNA sequencing

Total RNA from muscle samples (< 30 mg) was extracted using TRI reagent (Thermo Fisher Scientific, Waltham, MA, USA) and purified using the QIAGEN RNeasy Mini Kit (Qiagen, Hilden, Mettmann, Germany) according to the manufacturer’s instructions. Eluted RNA was stored at −80 °C until required for determination of total RNA and library processing. Total RNA was quantified using a Nanodrop 8000 (Thermo Fisher Scientific, Waltham, MA, USA), and RNA quality was assessed using an Agilent® Bioanalyser (Agilent, Santa Clara, CA, USA) with an average RIN score = 7.9, 260/280 = 2.01. RNA samples were then diluted to 20 ng·μL^−1^ using RNase-free water.

Libraries were constructed from 100 ng of total RNA with Poly-A tail enrichment of mRNA using NEBNext® Ultra™ II RNA Library Prep Kit for Illumina® with Agencourt AMPureXP Sample Purification Beads (Beckman Coulter. Wycombe, UK) as per manufacturers’ guidelines, by Bart’s and the London Genome Centre at Queen Mary, University of London. The resultant-barcoded libraries were sequenced on an Illumina NextSeq 2000 using 2 × 50 bp paired-end sequencing. An average of 36 million paired-end reads was achieved per sample post-trimming and alignment as described below.

FastQ files were imported to Partek® Flow® Genomic Analysis Software (Partek Inc. Missouri, USA) for pipeline processing. Pre-alignment QA/QC was performed on all reads prior to read trimming (Online Resource 1), before quality score trimming of reads with a Phred-score less than 20. STAR alignment 2.7.8a was then used to align trimmed reads to the human genome (GrCh38). Aligned reads were then quantified to the Ensembl transcriptome annotation model associated with human genome (hg) transcripts release 99. Post-alignment, QC reports are provided in Online Resource 2. Filtered raw counts were used for normalisation and differential analysis with DESeq2 through Partek® Flow® (Love et al. 2014). Gene transcripts were considered significantly different between groups when the false discovery rate (FDR) *q* < 0.05 and fold change (FC) > 1.5. To ensure robustness to the statistical framework, we additionally analysed the data using lmerseq (Vestal et al. 2022), which implements linear mixed-effects models appropriate for within-subject RNA-seq designs. Volcano plot was generated in R studio (version 2023.06.1 + 524); principal component analysis (PCA) plot was generated in Partek Flow®. Correlation plot was created in GraphPad Prism v10 (GraphPad Software, San Diego, CA). Raw data can be found at the GEO accession GSE292369. Further data will be made available by the corresponding author upon reasonable request.

### Statistical analysis

While the primary outcome of the current study is the global mRNA expression, there are not available data in human trials to base the sample size on this outcome. Statistical analysis of the transcriptomics data was performed as described in the previous paragraph. Other results are expressed as mean ± standard deviation (SD). Normality was tested with the Shapiro–Wilk test; if sphericity was not met, a Greenhouse–Geisser correction was applied. A two-way repeated measures analysis of variance (RM-ANOVA, [2 × 5] for serum βHB, glucose, and EPO; [2 × 2] for muscle glycogen concentration; [2 × 7] for cardiorespiratory, RPE, and substrate utilisation measurements) was utilised. If significant main effects or interactions effects were observed, post hoc testing was used with Bonferroni´s correction, with multiplicity-adjusted *P* values reported when comparing KME to PLA at respective time points. Significance was considered at *P* ≤ 0.05, and partial eta square ($\eta_{p}^{2}$: small effect = < 0.05; moderate effect = 0.05–0.14; large effect = > 0.14) was calculated to provided effect sizes if significant interactions occurred. A paired *t*-test was used to determine differences in total area under the curve (tAUC, for BHB, glucose, and EPO) (Narang et al. 2020) and deltas of skeletal muscle glycogen. All data were analysed in SPSS v29 (IBM Corp, Armonk, NY, USA). Figures were produced using GraphPad Prism v10 (GraphPad Software, San Diego, CA).

## Results

### Physiological and metabolic responses

As per experimental design, participants exercised at ~ 63% of V̇O_2max_ in both conditions. There were no significant interactions or condition effects for HR, RPE, V̇O_2_, V̇CO_2_, respiratory exchange ratio (RER), CHO oxidation, FAT oxidation, and lactate concentrations (*P* > 0.050). There was an increase in V̇O_2_, V̇CO_2_, HR, RPE, CHO oxidation, FAT oxidation, and lactate concentrations during exercise (*P* < 0.001, Table 1).

**Table 1:** Measures of physiological and metabolic responses during a 90-min cycling at 60% of V̇O_2max_

	Rest	12–15 min	27–30 min	42–45 min	57–60 min	72–75 min	87—90 min	*P*
V̇O_2_ (L·min^−1^)								
KME	0.47 ± 0.15	2.23 ± 0.16	2.29 ± 0.17	2.29 ± 0.18	2.33 ± 0.22	2.26 ± 0.17	2.37 ± 0.23	T*C, *P* = 0.288 **T, *P* < 0.001** C, *P* = 0.640
PLA	0.52 ± 0.14	2.31 ± 0.23	2.33 ± 0.19	2.37 ± 0.22	2.42 ± 0.22	2.41 ± 0.21	2.42 ± 0.26	
V̇CO_2_ (L·min^−1^)								
KME	0.44 ± 0.14	2.08 ± 0.17	2.11 ± 0.20	2.09 ± 0.20	2.14 ± 0.27	2.05 ± 0.23	2.14 ± 0.29	T*C, *P* = 0.674 **T, *P* < 0.001** C, *P* = 0.209
PLA	0.46 ± 0.10	2.15 ± 0.24	2.15 ± 0.21	2.15 ± 0.22	2.16 ± 0.23	2.13 ± 0.21	2.14 ± 0.30	
RER (AU)								
KME	0.93 ± 0.07	0.93 ± 0.02	0.92 ± 0.03	0.91 ± 0.02	0.92 ± 0.04	0.91 ± 0.04	0.90 ± 0.05	T*C, *P* = 0.473 T, *P* = 0.112 C, *P* = 0.231
PLA	0.90 ± 0.08	0.93 ± 0.05	0.92 ± 0.06	0.91 ± 0.04	0.89 ± 0.04	0.88 ± 0.04	0.88 ± 0.06	
HR (bpm)								
KME	67 ± 11	135 ± 18	149 ± 13	153 ± 14	149 ± 14	150 ± 15	151 ± 13	T*C, *P* = 0.441 **T, *P* < 0.001** C, *P* = 0.692
PLA	68 ± 8	142 ± 7	149 ± 9	151 ± 12	149 ± 10	151 ± 11	153 ± 13	
RPE (AU)								
KME	6 ± 0	9 ± 2	11 ± 1	12 ± 1	12 ± 1	13 ± 1	14 ± 1	T*C, *P* = 0.528 **T, *P* < 0.001** C, *P* = 0.321
PLA	6 ± 0	9 ± 1	11 ± 2	12 ± 1	12 ± 1	13 ± 2	13 ± 2	
CHO oxidation (g·min^−1^)								
KME	0.46 ± 0.21	2.13 ± 0.30	2.11 ± 0.41	2.02 ± 0.35	2.11 ± 0.52	1.96 ± 0.51	2.01 ± 0.66	T*C, *P* = 0.403 **T, *P* < 0.001** C, *P* = 0.535
PLA	0.40 ± 0.12	2.20 ± 0.55	2.15 ± 0.57	2.05 ± 0.41	1.93 ± 0.47	1.84 ± 0.44	1.84 ± 0.71	
FAT oxidation (g·min^−1^)								
KME	0.04 ± 0.05	0.22 ± 0.11	0.26 ± 0.14	0.29 ± 0.13	0.28 ± 0.16	0.30 ± 0.18	0.33 ± 0.22	T*C, *P* = 0.401 **T, *P* < 0.001** C, *P* = 0.528
PLA	0.09 ± 0.09	0.24 ± 0.22	0.26 ± 0.24	0.31 ± 0.18	0.38 ± 0.20	0.41 ± 0.21	0.42 ± 0.28	
Lactate (mmol·L^−1^)								
KME	2.15 ± 0.42	3.74 ± 0.67	3.18 ± 0.90	2.93 ± 1.10	2.50 ± 0.91	2.33 ± 0.81	2.04 ± 0.73	T*C, *P* = 0.084 **T, *P* < 0.001** C, *P* = 0.506
PLA	2.06 ± 0.32	3.69 ± 0.89	3.04 ± 1.09	2.83 ± 1.26	2.58 ± 1.21	2.63 ± 1.15	2.66 ± 1.04	

Participants (*n* = 9) received in a randomised order either KME (0.5 g·min^−1^) or PLA at 45 min. Resting and exercise data are means ± SD. Bold indicates P < 0.050 for main effect of time

*V̇O*_*2*_ volume of oxygen uptake, *V̇CO*_*2*_ volume of exhaled carbon dioxide, *RER* respiratory exchange ratio, *HR* heart rate, *RPE* rating of perceived exertion, *CHO* carbohydrate, *Fat* fat, *AU* arbitrary units, *bpm* beats per minute

### Blood/serum parameters

**Fig. 2:**
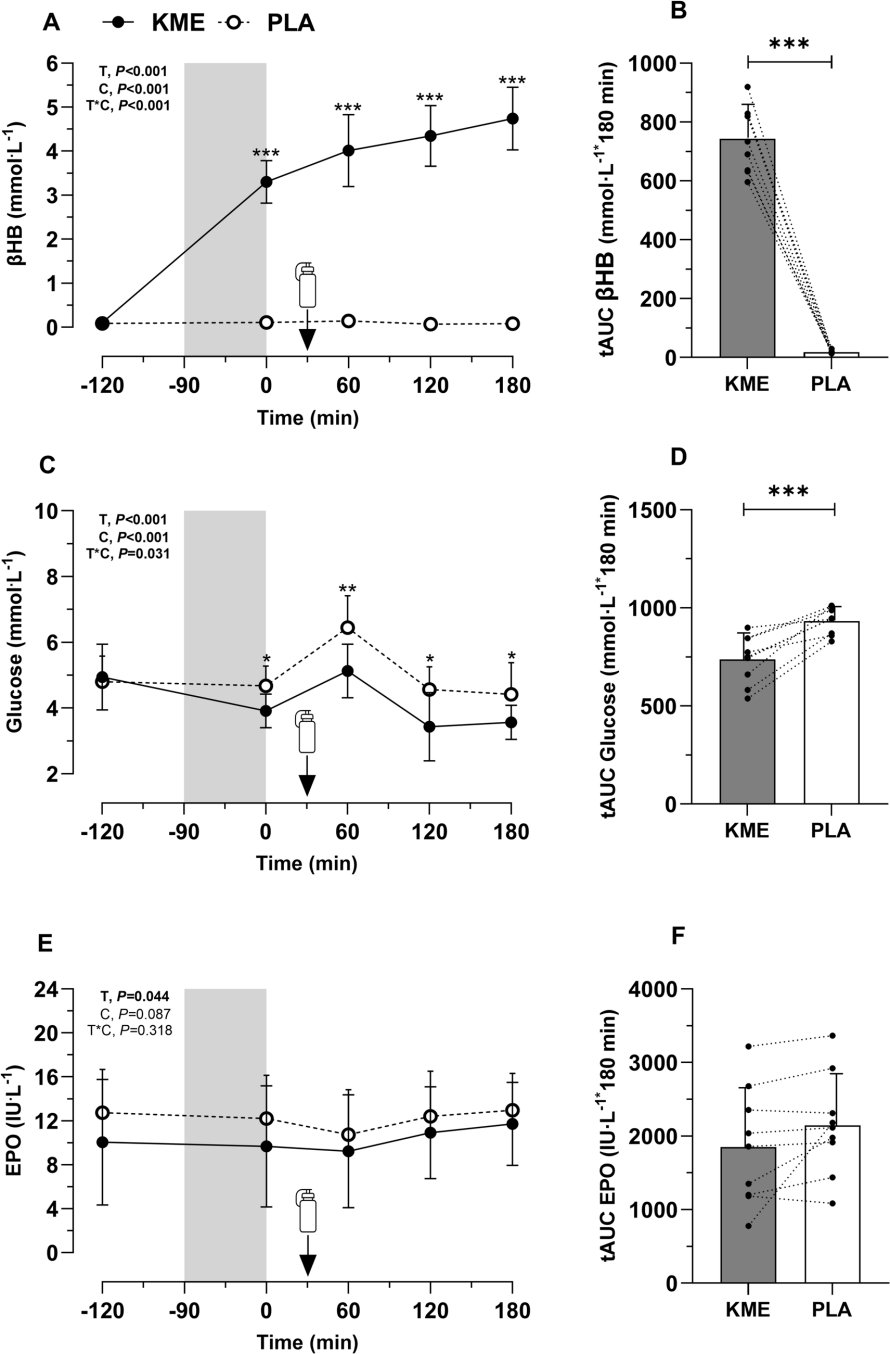
Blood/serum parameters. β-Hydroxybutyrate (βHB) (**A**), glucose (**C**), erythropoietin (EPO) concentrations (**E**) across the experimental trials incorporating ketone monoester (KME) or placebo (PLA) drink ingestion in healthy men during 90-min exercise at 60% of V̇O_2max_ (grey shade) and 180-min recovery. Carbohydrate and protein recovery drink ingestion time is indicated within panels with a bidon icon. Total area under the curve for βHB (**B**), glucose (**D**), and EPO (**F**). Data are presented as mean ± SD, * P < 0.050, ** P < 0.010, *** P < 0.001 for within time point comparison in panels **A**, **C** and **E**, and for condition comparison in panels **B**, **D** and **F**

βHB concentration showed a main effect of condition (*P* < 0.001), time (*P* < 0.001), and an interaction (*P* < 0.001, $\eta_{p}^{2}$ = 0.95—large—) **(**Fig. 2A**)**. Serum βHB PRE concentrations were ~ 0.1 mM in both conditions (*P* = 0.179). βHB values remained at ~ 0.1 mM throughout PLA, but βHB increased to 3.3 ± 0.5 mM immediately after exercise completion in KME. βHB concentrations remained consistently elevated across the post-exercise recovery period (range: ~ 3.3 to 4.7 mM, *P* < 0.001 at all-time points vs PLA). The tAUC for serum βHB was significantly higher for KME versus PLA (*P* < 0.001, Fig. 2B).

Glucose concentration showed a main effect of condition (*P* < 0.001), time (*P* < 0.001) and a significant interaction (*P* = 0.031, $\eta_{p}^{2}$ = 0.28—large—) (Fig. 2C**)**. Serum glucose PRE concentration was similar in both conditions (KME = 4.9 ± 1.0 mM, PLA = 4.8 ± 0.8 mM; *P* = 0.544). However, throughout the post-exercise period, glucose concentration was higher in PLA compared to KME (*P* < 0.050), resulting in a tAUC lower in KME (*P* < 0.001, Fig. 2D).

EPO concentration showed a time effect (*P* = 0.044), but no main effect of condition (*P* = 0.087), or interaction (*P* = 0.318, Fig. 2E**)**. Serum EPO concentration immediately after exercise was significantly higher compared to the 60-min time point (*P* = 0.029). No significant differences were observed between other time points. The tAUC for EPO was not significantly different between the conditions (*P* = 0.097, Fig. 2F).

### Glycogen

**Fig. 3:**
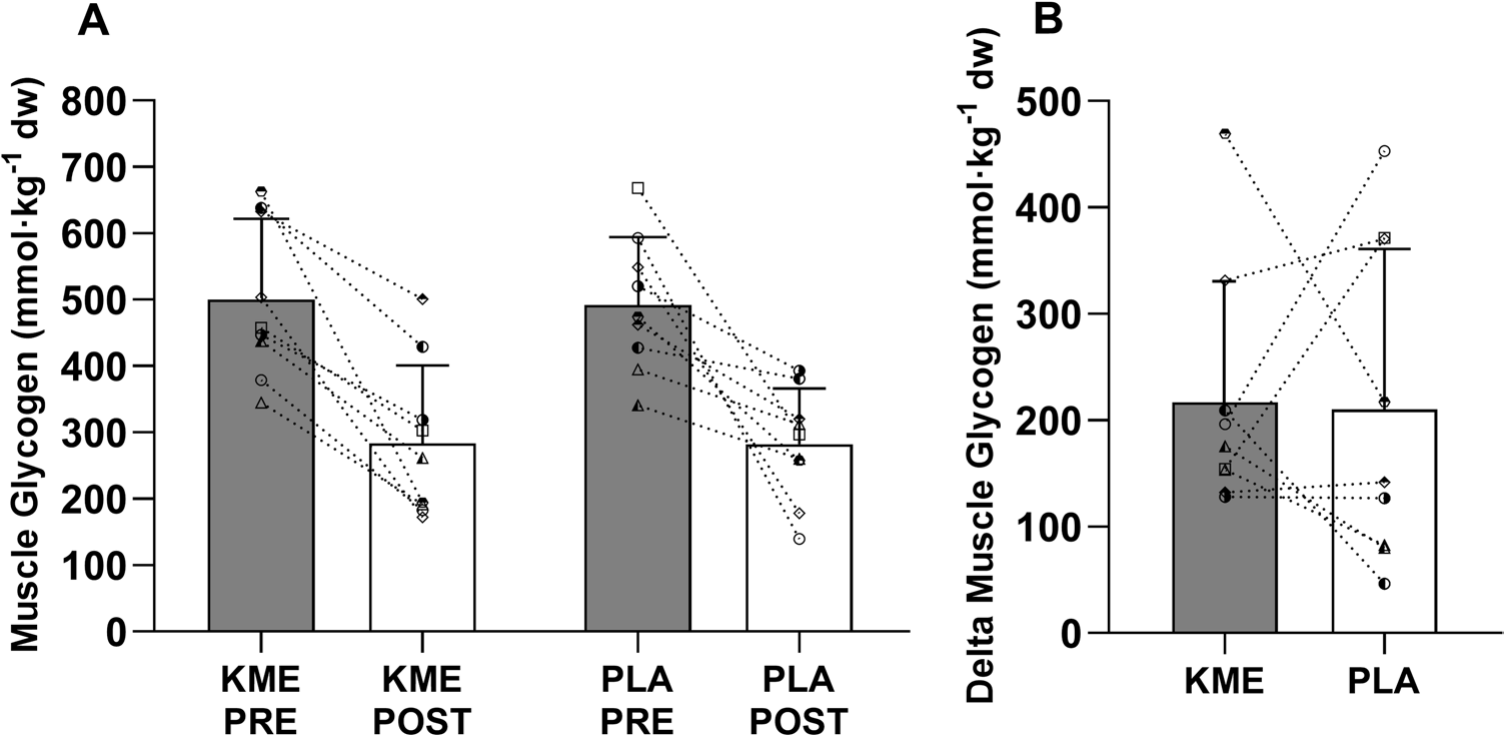
Muscle glycogen concentration. PRE and 180-min POST exercise muscle glycogen concentrations (**A**). Delta muscle glycogen concentrations (**B**). Data are presented as mean ± SD. A unique symbol is used to show values of each individual. Significant main effect of time (*P* < 0.001) for PRE vs POST muscle glycogen concentrations

PRE muscle glycogen concentrations did not differ between conditions (KME PRE: 501 ± 118 mmol·kg^-1^, PLA PRE: 492 ± 102 mmol·kg^-1^, *P* = 0.884, Fig. 3A) and were in accordance with predicted values based on diet and participant characteristics (Areta and Hopkins 2018). There was a main effect of time (*P* < 0.001) but no effect of condition (*P* = 0.889) or interaction (*P* = 0.907). There was a significant decrease in KME POST of 284 ± 117 mmol·kg^-1^ and PLA POST of 282 ± 184 mmol·kg^-1^ (*P* < 0.001), showing no difference between conditions (*P* = 0.957). Accordingly, there was no difference in the PRE/POST Δ glycogen between conditions (*P* = 0.907), with reductions of −217 ± 113 mmol·kg^-1^ and −210 ± 151 mmol·kg^-1^ for the KME and PLA conditions, respectively (Fig. 3B).

### Transcriptome responses

**Fig. 4:**
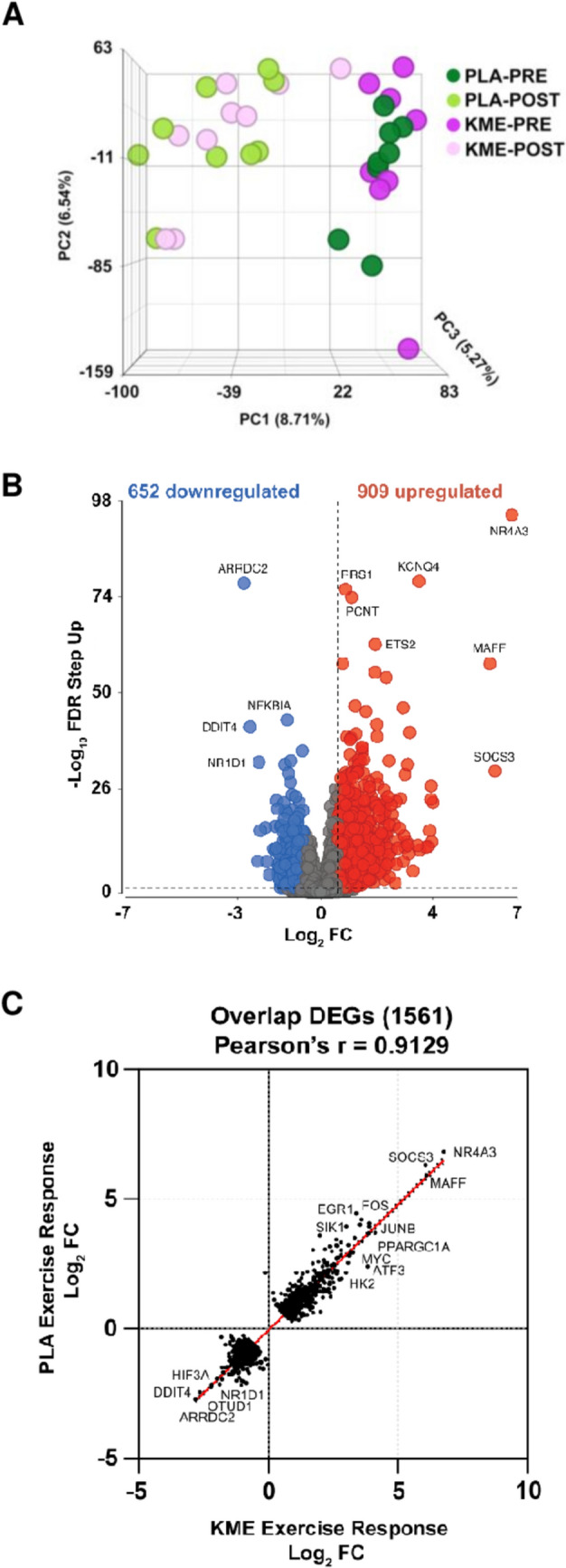
Skeletal muscle transcriptomic response between pre-exercise and 180 min post-exercise (90 min at 60% of VO2max) in physically active men who consumed a ketone monoester drink (KME) or a taste-matched placebo drink (PLA). Principal component analysis plot of skeletal muscle profiles comparing KME and PLA at PRE and POST exercise (**A**). Volcano plot illustrating the relationship between -log_10_FDR step-up and log_2_FC for exercise-induced skeletal muscle DEGs (**B**). Grey data points represent genes that are not statistically significant [-log_10_ FDR step-up (< 0.05) and log_2_FC < 1.5]. Top up-regulated and down-regulated DEGs are labelled. Correlation plot of Log_2_ fold changes including the overlapping up- and down-regulated DEGs that KME and PLA modulated similarly at POST (**C**)

The PCA plot revealed a clear separation of exercise-induced gene expression, but no separation between KME versus PLA, indicating no effect of condition **(**Fig. 4A**)**. Using an 0.05 FDR cutoff, we discovered that KME did not regulate the expression of any genes. Analyses using lmerseq (Vestal et al. 2022) were consistent with those obtained from DESeq2, with no additional differentially expressed genes identified at *q* < 0.05 (see Online Resource 3). Exercise significantly up-regulated and down-regulated the expression of 909 and 652 genes, respectively **(**Fig. 4B**).** To further examine the similarity in exercise-induced gene expression between conditions, we plotted the log_2_FC values of the DEGs in a correlation plot. The correlation plot illustrated a strong positive correlation in gene regulation between KME and PLA (*r* = 0.913, *P* < 0.001, Fig. 4C). We identified 1561 overlapped DEGs between conditions from which the most up-regulated (nuclear receptor subfamily 4 group A member 3, *NR4A3*; suppressor of cytokine signalling 3, *SOCS3*; MAF BZIP transcription factor F, *MAFF*; peroxisome proliferator-activated receptor gamma coactivator 1-alpha, *PPARGC1A*) and down-regulated genes (arresting domain containing 2, *ARRDC2*; hypoxia inducible factor 3 subunit alpha, *HIF3A*; DNA damage inducible transcript 4, *DDIT4*; nuclear receptor subfamily 1 group D member 1, *NR1D1*) highlight the influence of exercise alone on skeletal muscle transcriptome.

## Discussion

The main finding of this study was that despite ketone monoester supplementation elicited a large increase in circulating ketones throughout 180-min recovery post-exercise, there was no evidence of a condition effect on skeletal muscle transcriptome. We show for the first time that acute increases in ketonaemia do not affect the early skeletal muscle mRNA response, despite it being purported to enhance skeletal muscle recovery. Despite robust exercise-induced differentially expressed genes (1561, 9% of 16,898 genes measured), there is no evidence that KME supplementation modulated the skeletal muscle transcriptome early post-exercise. In addition, circulating erythropoietin and muscle glycogen levels were also unaffected by KME ingestion. These findings are contrary to our hypotheses.

Utilising a cohort of fit young men undergoing a 90-min bout of moderate-intensity aerobic exercise and a recovery period of 3 h with KME ingestion, this study evaluated for the first time the genome-wide transcriptomic response of skeletal muscle utilising state-of-the-art methodology for mRNA sequencing. We also sought to replicate the effects of KME on EPO and muscle glycogen dynamics reported by others (Vandoorne et al. 2017; Holdsworth et al. 2017; Evans et al. 2022; Poffé et al. 2023b), suggesting to mediate the KME adaptive response in muscle during recovery. Our study expands on recent findings in humans showing enhanced training adaptations during heavy training (Poffé et al. 2019), increased post-exercise EPO concentration (Evans et al. 2022), and increased angiogenesis (Poffé et al. 2023b) with KME supplementation, by providing insights into the effect on a key regulatory step in the modulation of skeletal muscle phenotype early post-exercise as it is mRNA expression.

Representing a significant step forward in the understanding of the physiological regulatory steps modulated by KME on skeletal muscle in humans, the stark lack of meaningful effects of KME on skeletal muscle transcriptome documented here was surprising and contrary to our hypothesis. Despite exercise showed a clear effect on gene expression (Fig. 4A, B), there was no clear segregation by condition on the principal component analysis of 16,898 expressed genes (Fig. 4 A). Moreover, we show a very strong positive correlation (*r* = 0.9129, *P* < 0.001) between both conditions post-exercise (Fig. 4C), indicating the robustness and reliability of our exercise paradigm, that was not meaningfully modulated by the condition. The modulation of the transcriptome by exercise aligns with previous reports, with the NR4A3 gene prominently represented as the most responsive gene in both conditions (Fig. 4C) (Pillon et al. 2020). The lack of condition effect contrasts with findings in non-human models of in vitro research. In myotubes widespread modulation of gene sets related to the citric acid cycle, oxidative phosphorylation, and amino acid metabolism, immunity and inflammation (Ruppert et al. 2021). In a model of unloading-induced atrophy in mice ketones modulated transcription of genes related to the ubiquitin–proteasome and autophagy-lysosome pathways, as well as genes of the tricarboxylic acid cycle, mitochondrial electron transport and pyruvate metabolism (Chen et al. 2022). Also, a model of ageing mice, documented modulation of 27 genes in skeletal muscle in response to ketones (Roberts et al. 2022). In humans, however, Poffé et al. (2023a, b) showed an increase in mRNA of eNOS and VEGF after 3-week ketone supplementation with concomitant training overload, which contrasts with our findings, but this is perhaps related to the lack of EPO changes in KME or the timing of the muscle biopsy sampling.

The expected increase in EPO concentration induced with KME was based on recent reports of different research protocols including acute infusion of ketones in resting individuals (Lauritsen et al. 2020), acute post-exercise KME ingestion (Evans et al. 2022), and chronic provision of KME during 3 weeks (Poffé et al. 2023b). Perhaps the timeframe of 3 h post-exercise in our study (Fig. 1) was insufficient to elicit detectable rises in EPO (Fig. 2, E and F), given that the other acute studies showing an increase of EPO by KME used protocols of ~ 4 to 7 h of elevated ketosis (Lauritsen et al. 2020; Evans et al. 2022). Similar to our results, Howard et al. (2024) observed no effect of KME immediately post-exercise in EPO concentrations. The lack of detectable EPO concentration increases may partially explain the lack of increase in angiogenesis-related mRNA genes and capillarisation by KME in muscle previously reported (Poffé et al. 2023b). It appears, then, that without changes in EPO, changes in circulating ketones alone may not be sufficient to elicit a response in skeletal muscle transcriptome. Despite the limited evidence suggesting the lack of an effect in muscle, KME did have a clear metabolic effect.

The reduction of blood glucose with KME is now a well-established metabolic response (Falkenhain et al. 2022), but this did not seem to have a significant effect on skeletal muscle glycogen. Even though the increase in circulating ketones has been shown to increase muscle glycogen resynthesis in non-human models (Takahashi et al. 2019), and in a hyperinsulinemic-clamp human model post-exercise (Holdsworth et al. 2017), other studies in humans under euglycemic conditions have reported no effect of hyperketonaemia on skeletal muscle glucose uptake (Lauritsen et al. 2020; Beylot et al. 1986; Walker et al. 1991). In agreement with our results, Vandoorne et al. (2017) showed no effect of KME on muscle glycogen resynthesis during 5 h post-exercise when an optimal diet for muscle glycogen resynthesis of 1 g·kg·h^−1^ carbohydrates and 0.3 g·kg·h^−1^ of protein was provided. Even when a diet with sub-optimal carbohydrate content was provided in our study (1 g·kg^−1^; Fig. 1), KME did not affect muscle glycogen levels post-recovery (Fig. 3, B). It is therefore likely that the metabolic effects of ketones on circulating glucose are related to regulation of the glycaemic response by the liver rather than increased uptake of glucose by the muscle (Geisler et al. 2019; Howard et al. 2023).

Given the effect of KME on human skeletal muscle protein phosphorylation state (Vandoorne et al. 2017; Poffé et al. 2023b), mTOR translocation (Hannaian et al. 2024), and protein synthesis (Hannaian et al. 2024; Nair et al. 1988), the lack of difference in the skeletal muscle transcriptome is surprising, though we cannot discard that ketones may directly, or through endocrine regulation, have the capacity to modulate the skeletal muscle adaptive response through other mechanisms. In addition, since the mRNA transcripts are dependent on the timing of muscle sampling (Kuang et al. 2022), the transcriptional response could be distinct at a different time point. Therefore, future research should investigate other possible levels of biological regulation of skeletal muscle phenotype, such as epigenetic (acetylation, methylation, and β-hydroxybutyrylation), intracellular signalling, and/or proteomic regulation.

## Conclusion

In conclusion, the observed data do not substantiate an effect of KME supplementation on the skeletal muscle transcriptome response to exercise, glycogen resynthesis, and EPO concentrations during early recovery. Collectively, the mechanistic and physiological data from this study do not support the idea that KME modulates early post-exercise recovery at the transcription level, at least when ingested acutely, and suggest that other levels of biological regulation should be investigated to further characterise the effect of KME on muscle.

## Supplementary Information

Below is the link to the electronic supplementary material.

Supplementary file1 (PDF 153 KB)

Supplementary file2 (PDF 201 KB)

Supplementary file3 (XLSX 14695 KB)

## Data Availability

The data that support the findings of this study are available from the corresponding author upon reasonable request.

## Abbreviations

AU: Arbitrary units
CHO: Carbohydrate
DEGs: Differentially expressed genes
ELISA: Enzyme-linked immunosorbent assay
eNOS: Endothelial nitric oxide synthase
EPO: Erythropoietin
FAT: Fat
FC: Fold change
FDR: False discovery rate
HR: Heart rate
KME: Ketone monoester
mRNA: Messenger ribonucleic acid
PCA: Principal component analysis
PLA: Placebo
PRO: Protein
RER: Respiratory exchange ratio
RIN: RNA integrity number
RM-ANOVA: Repeated measures analysis of variance
RPE: Rating of perceived exertion
tAUC: Total area under the curve
VEGF: Vascular endothelial growth factor
V̇O₂max: Maximal oxygen uptake
βHB: Beta-hydroxybutyrate
