# Supplementary material for: Acute nutritional ketosis during early recovery from aerobic exercise does not affect skeletal muscle transcriptomic response in humans
Source: Eur J Appl Physiol. 2025 Sep 17;126(2):1021–32. doi: 10.1007/s00421-025-05987-9 (PMC12948831; doi:10.1007/s00421-025-05987-9)
Supplement: Supplementary file 1 — Supplementary file1 (PDF 153 KB) [file 421_2025_5987_MOESM1_ESM.pdf]

Acute Nutritional Ketosis During Early Recovery From Aerobic Exercise Does Not Affect Skeletal Muscle Transcriptomic Response in Humans

European Journal of Applied Physiology

Erick Mosquera-Lopez[1], Julien Louis[1], Jason P. Edwards[1], Jamie Pugh[1], Mark R. Viggars[2], Daniel J. Owens[1], Jose L. Areta[1*]

[1] Liverpool John Moores University, Research Institute for Sport & Exercise Sciences (RISES), Liverpool, UK

[2] University of Florida, Department of Physiology and Aging, Gainesville, FL, USA

***Corresponding author:**
Jose L. Areta
Research Institute for Sport and Exercise Sciences, Liverpool John Moores University, Tom Reilly Building, Byrom St Campus, Liverpool L3 3AF, UK

j.l.areta@ljmu.ac.uk

Online Resource 1 Pre-alignment QA/QC report.

**Sample name**	**Subject**	**Condition**	**Time point**	**Total reads**	**Read length**	**Avg. read quality**	**% N**	**% GC**
GC-DO-11238-1-1-PLA-1_S19	1	PLA	PRE	37172791	50	39.41	0.0440753	50.31
GC-DO-11238-1-1-PLA-2_S32	1	PLA	POST	41588046	50	39.41	0.0439128	50.79
GC-DO-11238-1-2-KET-3_S7	1	KET	PRE	35152289	50	39.42	0.0443256	50.71
GC-DO-11238-1-2-KET-4_S20	1	KET	POST	36566448	50	39.42	0.0440844	50.52
GC-DO-11238-2-1-KET-5_S35	2	KET	PRE	26892634	50	39.43	0.0440903	50.75
GC-DO-11238-2-1-KET-6_S27	2	KET	POST	39893049	50	39.41	0.0439315	50.52
GC-DO-11238-2-2-PLA-7_S31	2	PLA	PRE	41941180	50	39.44	0.0437535	47.18
GC-DO-11238-2-2-PLA-8_S40	2	PLA	POST	44430002	50	39.42	0.0436923	50.29
GC-DO-11238-3-1-KET-9_S30	3	KET	PRE	39108029	50	39.42	0.0439564	50.74
GC-DO-11238-3-1-KET-10_S38	3	KET	POST	38876982	50	39.40	0.044035	50.72
GC-DO-11238-3-2-PLA-11_S24	3	PLA	PRE	30354783	50	39.43	0.0438172	49.55
GC-DO-11238-3-2-PLA-12_S23	3	PLA	POST	39622732	50	39.43	0.0442048	50.35
GC-DO-11238-4-1-KET-13_S11	4	KET	PRE	24783414	50	39.40	0.0443103	50.48
GC-DO-11238-4-1-KET-14_S28	4	KET	POST	36741315	50	39.42	0.0438731	50.59
GC-DO-11238-4-2-PLA-15_S25	4	PLA	PRE	36646048	50	39.41	0.0439543	50.50
GC-DO-11238-4-2-PLA-16_S17	4	PLA	POST	37145432	50	39.42	0.0439963	50.44
GC-DO-11238-6-1-KET-21_S37	6	KET	PRE	29746710	50	39.42	0.0437993	50.55
GC-DO-11238-6-1-KET-22_S21	6	KET	POST	39549339	50	39.41	0.0437687	50.58
GC-DO-11238-6-2-PLA-23_S12	6	PLA	PRE	36804814	50	39.42	0.0443088	51.07
GC-DO-11238-6-2-PLA-24_S3	6	PLA	POST	31053459	50	39.41	0.0445089	50.57
GC-DO-11238-7-1-KET-25_S2	7	KET	PRE	26613726	50	39.40	0.0438633	50.01
GC-DO-11238-7-1-KET-26_S34	7	KET	POST	33753015	50	39.40	0.0438276	50.64
GC-DO-11238-7-2-PLA-27_S10	7	PLA	PRE	39006619	50	39.42	0.0437558	50.04
GC-DO-11238-7-2-PLA-28_S16	7	PLA	POST	40464015	50	39.42	0.0439567	50.17

GC-DO-11238-8-1-KET-29_S29	8	KET	PRE	42136040	50	39.40	0.0436404	50.84
GC-DO-11238-8-1-KET-30_S39	8	KET	POST	31259754	50	39.44	0.043919	50.79
GC-DO-11238-8-2-PLA-31_S14	8	PLA	PRE	38244179	50	39.40	0.0436852	50.35
GC-DO-11238-8-2-PLA-32_S4	8	PLA	POST	32689420	50	39.43	0.0438746	50.16
GC-DO-11238-9-1-PLA-33_S5	9	PLA	PRE	34976344	50	39.41	0.0436681	50.52
GC-DO-11238-9-1-PLA-34_S22	9	PLA	POST	39610869	50	39.42	0.0440131	50.46
GC-DO-11238-9-2-KET-35_S15	9	KET	PRE	42152208	50	39.40	0.0442598	50.58
GC-DO-11238-9-2-KET-36_S33	9	KET	POST	38390674	50	39.42	0.0436588	50.75
GC-DO-11238-10-1-KET-37_S8	10	KET	PRE	35038939	50	39.41	0.0439346	50.81
GC-DO-11238-10-1-KET-38_S18	10	KET	POST	39613654	50	39.41	0.0438542	50.97
GC-DO-11238-10-2-PLA-39_S9	10	PLA	PRE	33489999	50	39.40	0.0439901	50.84
GC-DO-11238-10-2-PLA-40_S36	10	PLA	POST	30048834	50	39.42	0.0442844	50.97
