# Supplementary material for: Acute nutritional ketosis during early recovery from aerobic exercise does not affect skeletal muscle transcriptomic response in humans
Source: Eur J Appl Physiol. 2025 Sep 17;126(2):1021–32. doi: 10.1007/s00421-025-05987-9 (PMC12948831; doi:10.1007/s00421-025-05987-9)
Supplement: Supplementary file 2 — Supplementary file2 (PDF 201 KB) [file 421_2025_5987_MOESM2_ESM.pdf]

Acute Nutritional Ketosis During Early Recovery From Aerobic Exercise Does Not Affect Skeletal Muscle Transcriptomic Response in Humans

European Journal of Applied Physiology

Erick Mosquera-Lopez[1], Julien Louis[1], Jason P. Edwards[1], Jamie Pugh[1], Mark R. Viggars[2], Daniel J. Owens[1], Jose L. Areta[1*]

[1] Liverpool John Moores University, Research Institute for Sport & Exercise Sciences (RISES), Liverpool, UK

[2] University of Florida, Department of Physiology and Aging, Gainesville, FL, USA

***Corresponding author:**
Jose L. Areta
Research Institute for Sport and Exercise Sciences, Liverpool John Moores University, Tom Reilly Building, Byrom St Campus, Liverpool L3 3AF, UK

j.l.areta@ljmu.ac.uk

Online Resource 2 Post-alignment QA/QC report

Sample name	Total reads	Total alignments	Aligned %	Total unaligned	Unaligned %	Total unique singleton	Unique singleton	Total unique paired	Unique paired	Total non-unique paired	Non-unique paired	Total non-unique singleton	Non-unique singleton	Coverage	Avg. coverage depth	Avg. length	Avg. quality	%GC
GC-DO-11238-1-1-PLA-1_S19	37172402	77870848	97.30	1004426	2.70	35	9.42E-05	34222195	92.063448	1945743	5.23	3	4.17	29.87	49.99	39.48	50.20	4.17
GC-DO-11238-1-1-PLA-2_S32	41587563	86938506	97.36	1098779	2.64	46	1.11E-04	38413643	92.3681029	2075088	4.99	7	4.64	29.99	49.99	39.48	50.70	4.64
GC-DO-11238-1-2-KET-3_S7	35152011	73561922	97.27	957979	2.73	34	9.67E-05	32380438	92.1154639	1813558	5.16	2	3.73	31.57	49.99	39.48	50.58	3.73
GC-DO-11238-1-2-KET-4_S20	36566012	76393261	97.40	952474	2.60	43	1.18E-04	33731819	92.2491055	1881672	5.15	4	3.80	32.17	49.99	39.49	50.39	3.80
GC-DO-11238-2-1-KET-5_S35	26892426	56018781	97.49	673795	2.51	20	7.44E-05	24966063	92.8367824	1252545	4.66	3	3.60	24.93	49.99	39.49	50.62	3.60
GC-DO-11238-2-1-KET-6_S27	39892793	83056236	97.44	1020720	2.56	25	6.27E-05	36980078	92.6986436	1891969	4.74	1	4.20	31.67	49.99	39.48	50.40	4.20

GC-DO-11238-2-2-PLA-7_S31	41940892	89863364	97.50	1046691	2.50	18	4.29E-05	38185229	91.0453431	2708952	6.46	2	4.50	32.06	49.99	39.50	47.25	4.50
GC-DO-11238-2-2-PLA-8_S40	44429618	92939807	97.68	1030667	2.32	46	1.04E-04	41207690	92.7482429	2191212	4.93	3	4.65	32.02	49.99	39.48	50.23	4.65
GC-DO-11238-3-1-KET-9_S30	39107784	82209565	97.62	932027	2.38	27	6.90E-05	36017850	92.0989284	2157880	5.52	0	4.00	32.89	49.99	39.48	50.64	4.00
GC-DO-11238-3-1-KET-10_S38	38876636	81141164	97.49	975010	2.51	28	7.20E-05	35962844	92.5050305	1938747	4.99	7	3.97	32.75	49.99	39.48	50.62	3.97
GC-DO-11238-3-2-PLA-11_S24	30354697	64027753	97.71	695586	2.29	11	3.62E-05	27782264	91.5254203	1876835	6.18	1	3.32	30.98	49.99	39.50	49.41	3.32
GC-DO-11238-3-2-PLA-12_S23	39622312	82889162	97.47	1002152	2.53	54	1.36E-04	36527130	92.1882852	2092971	5.28	5	4.17	31.82	49.99	39.50	50.23	4.17
GC-DO-11238-4-1-KET-13_S11	24783214	51957442	96.93	760916	3.07	23	9.28E-05	22633269	91.3249952	1389003	5.60	3	4.10	20.26	49.99	39.48	50.37	4.10

GC-DO-11238-4-1-KET-14_S28	36741108	77538672	97.41	952636	2.59	20	5.44E-05	33785306	91.9550548	2003145	5.45	1	4.22	29.41	49.99	39.48	50.62	4.22
GC-DO-11238-4-2-PLA-15_S25	36645859	77378657	97.52	909694	2.48	23	6.28E-05	33636771	91.7887366	2099369	5.73	2	4.15	29.88	49.99	39.47	50.43	4.15
GC-DO-11238-4-2-PLA-16_S17	37144935	78200510	97.38	974079	2.62	50	1.35E-04	34165316	91.9783976	2005488	5.40	2	4.02	31.16	49.99	39.49	50.40	4.02
GC-DO-11238-6-1-KET-21_S37	29746472	62473811	97.46	754115	2.54	25	8.40E-05	27410410	92.1467595	1581919	5.32	3	3.37	29.74	49.99	39.48	50.44	3.37
GC-DO-11238-6-1-KET-22_S21	39548984	82681877	97.46	1005895	2.54	34	8.60E-05	36553332	92.4254641	1989721	5.03	2	4.07	32.56	49.99	39.48	50.48	4.07
GC-DO-11238-6-2-PLA-23_S12	36804532	76847675	97.09	1070039	2.91	31	8.42E-05	33872238	92.032791	1862223	5.06	1	3.81	32.28	49.99	39.48	50.90	3.81
GC-DO-11238-6-2-PLA-24_S3	31053344	64895270	97.45	791003	2.55	18	5.80E-05	28677007	92.3475649	1585316	5.11	0	3.53	29.40	49.99	39.48	50.46	3.53

GC-DO-11238-7-1-KET-25_S2	26613500	55810434	97.14	759913	2.86	18	6.76E-05	24412115	91.7283146	1441453	5.42	1	4.50	19.86	49.99	39.48	49.88	4.50
GC-DO-11238-7-1-KET-26_S34	33752728	70505690	97.53	833826	2.47	26	7.70E-05	31189826	92.40683	1729048	5.12	2	4.02	28.12	49.99	39.47	50.53	4.02
GC-DO-11238-7-2-PLA-27_S10	39006257	81809180	97.49	980437	2.51	31	7.95E-05	35839970	91.8826177	2185815	5.60	4	4.08	32.14	49.99	39.48	49.92	4.08
GC-DO-11238-7-2-PLA-28_S16	40463548	84511865	97.38	1059104	2.62	46	1.14E-04	37213956	91.9690878	2190437	5.41	5	4.11	32.92	49.99	39.49	50.01	4.11
GC-DO-11238-8-1-KET-29_S29	42135796	88115989	97.47	1066848	2.53	24	5.70E-05	38951535	92.4428602	2117386	5.03	3	4.12	34.27	49.99	39.47	50.74	4.12
GC-DO-11238-8-1-KET-30_S39	31259526	65151541	97.58	756012	2.42	21	6.72E-05	28970051	92.6759126	1533441	4.91	1	3.57	29.25	49.99	39.50	50.66	3.57
GC-DO-11238-8-2-PLA-31_S14	38243784	80667895	97.30	1032237	2.70	33	8.63E-05	34954250	91.3985133	2257262	5.90	2	3.68	35.08	49.99	39.48	50.25	3.68

GC-DO-11238-8-2-PLA-32_S4	32689087	68435982	97.44	837166	2.56	30	9.18E-05	30038670	91.8920434	1813218	5.55	3	3.56	30.80	49.99	39.49	50.00	3.56
GC-DO-11238-9-1-PLA-33_S5	34975975	73255280	97.43	898947	2.57	34	9.72E-05	32212430	92.0987335	1864560	5.33	4	3.87	30.33	49.99	39.49	50.37	3.87
GC-DO-11238-9-1-PLA-34_S22	39610482	82870415	97.57	961975	2.43	37	9.34E-05	36609028	92.4225764	2039439	5.15	3	3.91	33.95	49.99	39.48	50.32	3.91
GC-DO-11238-9-2-KET-35_S15	42151760	88429176	97.39	1099433	2.61	40	9.49E-05	38777328	91.9945644	2274955	5.40	4	4.07	34.77	49.99	39.47	50.44	4.07
GC-DO-11238-9-2-KET-36_S33	38390432	80510396	97.63	908306	2.37	16	4.17E-05	35472314	92.3988404	2009792	5.24	4	3.93	32.80	49.99	39.48	50.63	3.93
GC-DO-11238-10-1-KET-37_S8	35038590	73280314	97.28	952070	2.72	32	9.13E-05	32222528	91.96297	1863957	5.32	3	3.54	33.13	49.99	39.47	50.69	3.54
GC-DO-11238-10-1-KET-38_S18	39613097	83097568	97.36	1046504	2.64	44	1.11E-04	36497711	92.1354647	2068831	5.22	7	3.83	34.75	49.99	39.47	50.91	3.83

GC-DO-11238-10-2-PLA-39_S9	33489688	69843656	97.20	938040	2.80	34	1.02E-04	30882043	92.2135883	1669567	4.99	4	3.80	29.42	49.99	39.47	50.74	3.80
GC-DO-11238-10-2-PLA-40_S36	30048603	62839529	97.55	735158	2.45	25	8.32E-05	27769523	92.4153546	1543897	5.14	0	3.50	28.73	49.99	39.48	50.88	3.50
